# Emergence of SARS‐CoV‐2 Omicron variant and strategies for tackling the infection

**DOI:** 10.1002/iid3.733

**Published:** 2022-11-07

**Authors:** Yan Wu, Yiyin Long, Feng Wang, Wei Liu, Yuliang Wang

**Affiliations:** ^1^ Department of Clinical Laboratory Medicine Tianjin TEDA Hospital Tianjin China; ^2^ Tianjin Institute of Urology The Second Hospital of Tianjin Medical University Tianjin China; ^3^ Department of Genetics, School of Basic Medical Sciences Tianjin Medical University Tianjin China; ^4^ Tianjin Children's Hospital Children's Hospital of Tianjin University Tianjin China

**Keywords:** mutations, Omicron, severe acute respiratory syndrome coronavirus 2, transmissibility, vaccination

## Abstract

**Introduction:**

Nowadays, emerging SARS‐CoV‐2 Omicron, the novel highly mutated VOC, has quickly spread as the dominant variant in over 190 countries worldwide through the first part of 2022, which is influencing the infectivity, transmissibility, pathogenicity, and severity of COVID‐19 pandemic. Additionally, clinical cases and experimental studies have reported that Omicron variant likely leads to weakened immune protection elicited by infection, antibody therapies, and vaccines. The new wave, from late February, 2022, was escalated abruptly by higher levels of transmission of Omicron BA.2 sublineage in China.

**Methods and Results:**

Following a systematic database search, this review summarizes the salient features of Omicron sublineages, and their impact on transmissibility, disease severity as well as the efficacy of the available vaccines and treatment against the Omicron.

**Conclusion:**

We hope this study will provide a scientific reference for alleviating the burden of COVID‐19.

## Graphical abstract

SARS‐CoV‐2 Omicron variant is influencing the infectivity, transmissibility, pathogenicity, and severity of the COVID‐19 pandemic. Omicron variant displayed a reduction of immune protection against infection and vaccine. This article highlights the salient feature and mutation of Omicron sublineages, transmissibility, severity, and effectiveness of vaccination/treatment against the Omicron variant.

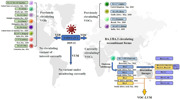

## 1 INTRODUCTION

**Figure 1:**
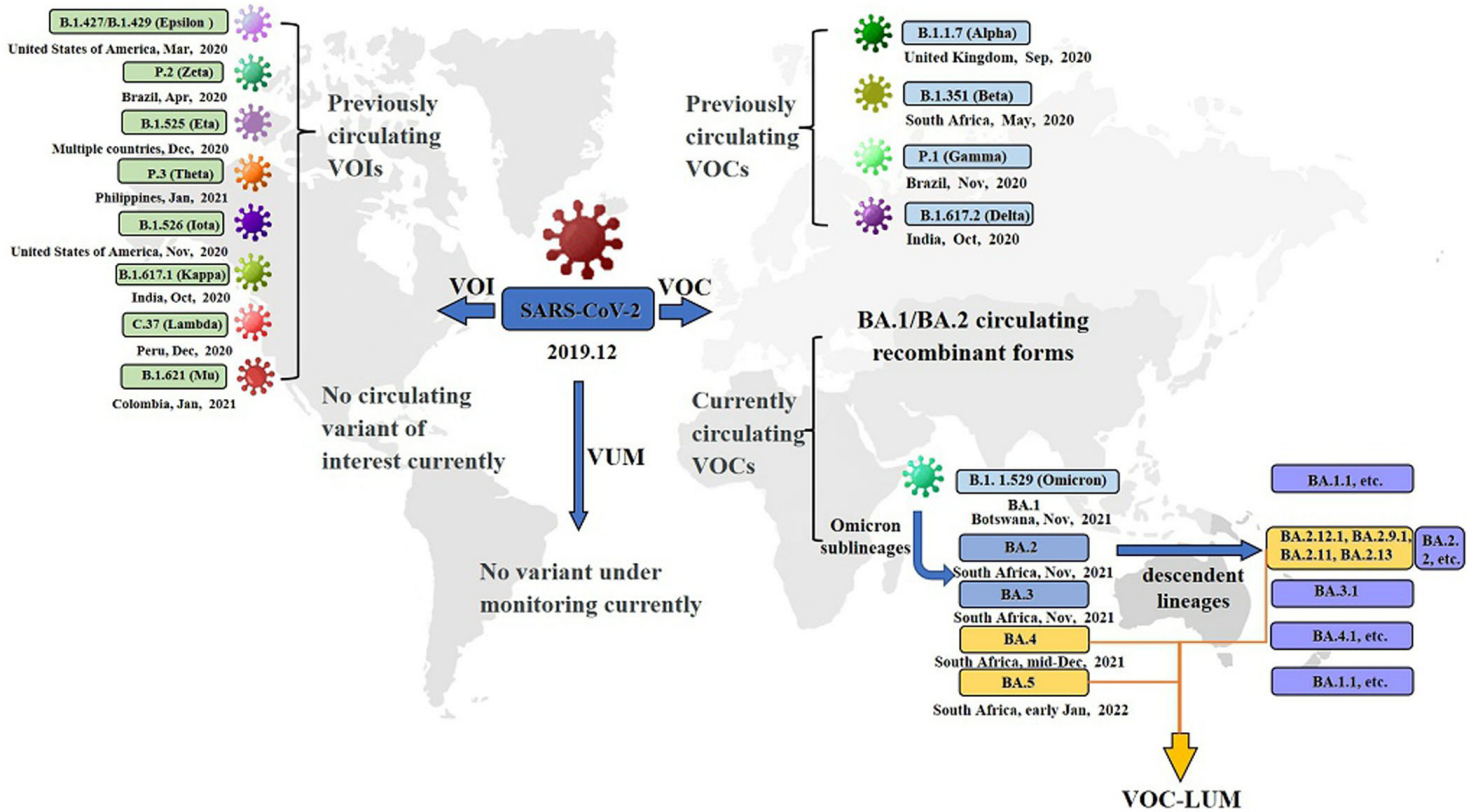
Global SARS‐CoV‐2 VOC, VOC‐LUM, VOI, and VOM. VOC, variants of concern; VOC‐LUM, VOC lineages under monitoring; VOI, variants of interest, VUM: variants under monitoring.

**Figure 2:**
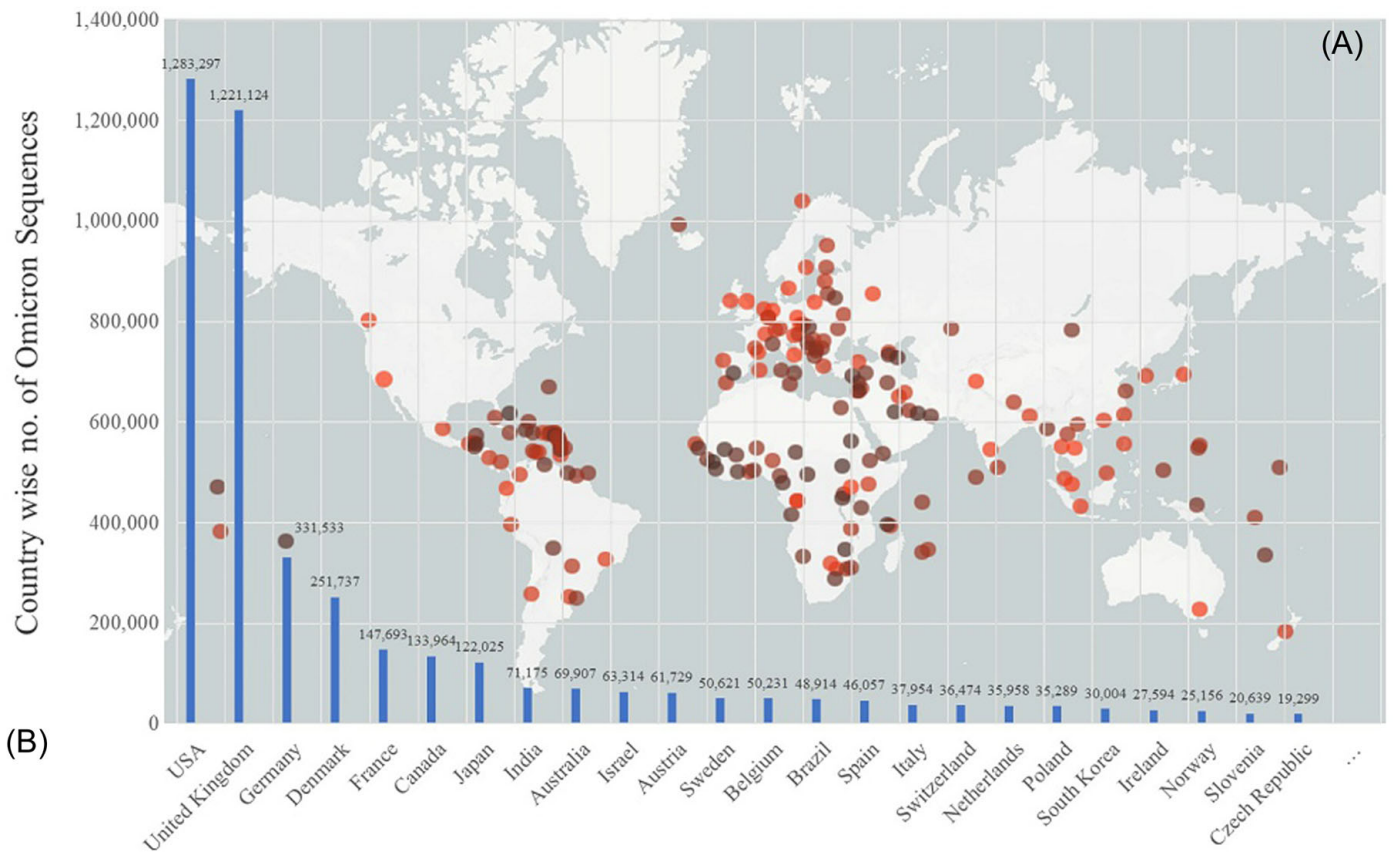
Emergence of Omicron worldwide. (A) Map of tracked Omicron variant occurrence. (B) Countries using sequence data from GISAID, accessed on July 6, 2022. GISAID, global initiative on sharing all influenza data.

The COVID‐19, highly contagious disease, has led to more than 603,711,760 confirmed cases and 6,484,136 cumulative fatalities caused by SARS‐CoV‐2.^1^ Since the outbreak of global pandemic, multiple types of variants have been confirmed, based on the characteristics of the infectivity/transmissibility with SARS‐CoV‐2, the severity of COVID‐19‐associated disease, or the impact on vaccine interventions, therapeutic medicines and diagnostics (Figure 1).^2^, ^3^ Recently, emerging Omicron sublineages, variable vaccination coverage across and within countries, and uncertainty about the protection of vaccine brought complexities to the recovery plans.^4^, ^5^ Present epidemiological update shows that rises in the COVID‐19 cases have coincided with the 3,881,444 SARS‐CoV‐2 Omicron genome sequences shared by more than 190 countries/territories around the world with unprecedented speed, leading to another COVID‐19 case spike so as to aggravate the COVID‐19 pandemic (Figure 2).^6^ This review summarizes the salient features of Omicron sublineages, and their impact on transmissibility, disease severity as well as the efficacy of the available vaccines and treatment against the Omicron, hoping to provide a scientific reference for alleviating the burden of COVID‐19.

## 2 EMERGING OF OMICRON SUBLINEAGES

**Figure 3:**
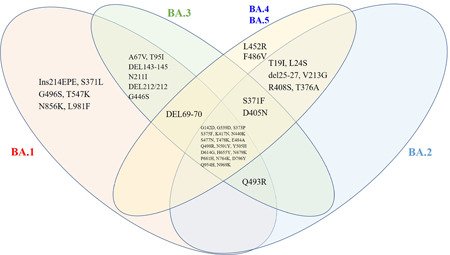
Using Venn diagram to represent spike mutations of Omicron sublineages

On November 11, 2021, a new COVID‐19 case was initially discovered in the Botswana which was subsequently increased in Gauteng province, South Africa.^7^ On 22 November 2021, these genome sequence of this novel lineage (B.1.1.529), was designated in global initiative on sharing all influenza database.^6^ Subsequent surveillance was revealed that B.1.1.529 harbored up to a total of 60 mutations (substitutions/deletions/insertions) throughout its genome compared to the ancestral wild type strain (WT).^8^ On November 26, B.1.1.529 was promptly categorized as the fifth VOC and named it Omicron by World Health Organization (WHO), and was subsequently causing great public health concerns.^9^ So far, the mutating Omicron has evolved into five distinct sublineages, the standard sublineage of Omicron is designated as B.1.1.529.1 (BA.1), whereas the other four sublineages of Omicron are referred to as BA.2, BA.3, BA.4,and BA.5 from the same place based on the different mutational profiles in genes encoding SARS‐CoV‐2 spike (S) protein of in the sequenced samples.^10^, ^11^ As the most critical structural protein of SARS‐CoV‐2, S protein is not only required in viral attachment, fusion, and entry, but a primary antigenic target for the development of therapeutic antibodies as well as available vaccines.^12^, ^13^ Compared to the WT, it is inferred that there are 37S mutations (including 30 amino acid substitutions, 1 insertion and 6 deletions) in BA.1, 31S mutations (including 28 amino acid substitutions and 3 deletions) in BA.2, 33S mutations (including 27 amino acid substitutions and 6 deletions) in BA.3, and 34S mutations (including 29 amino acid substitutions and 5 deletions) in BA.4/5.^14^, ^15^, ^16^ In particular, 15 mutations accumulated in the receptor‐binding domain of Omicron BA.1, whose binding with host ACE2 contributes to strengthen infectivity/transmissibility as well as to reduce the protection afforded by vaccines in current use or prior natural infections induced humoral immunity (Figure 3).^17^, ^18^ Consequently, the evolution of Omicron variant leads to high global public health concerns and a broadly socioeconomic burden.^18^

**Figure 4:**
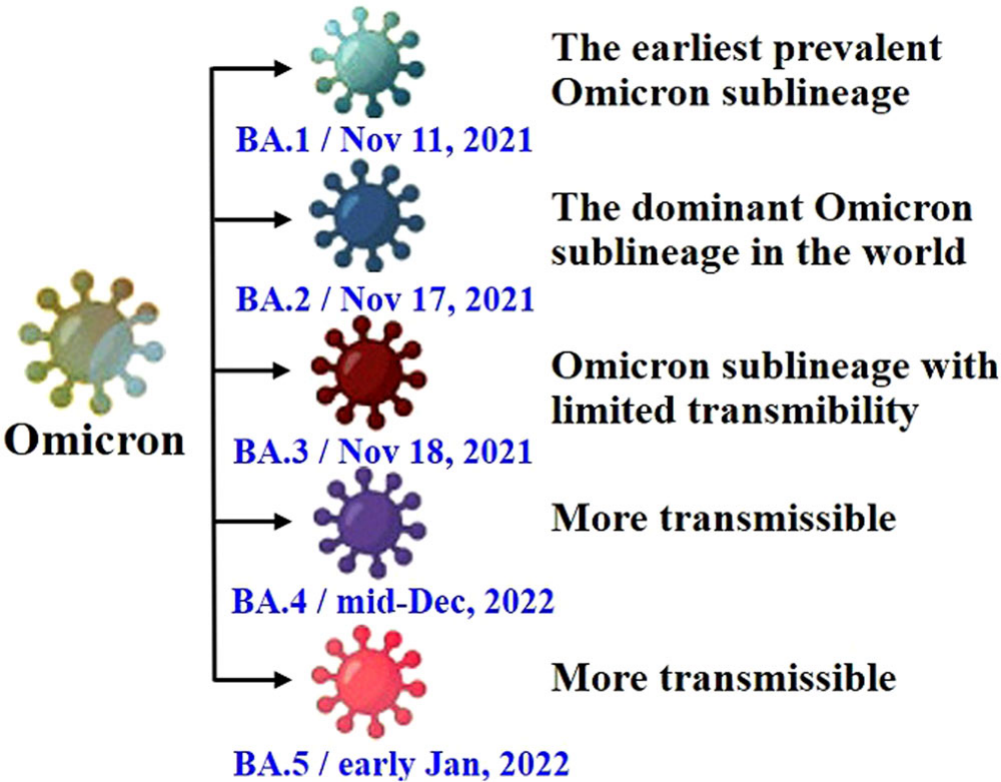
The spreading of Omicron five sublineages

During the early phase of the Omicron's spread, BA.1 spread to over 160 nations and regions.^19^ However, the number of detected BA.2 genomes increased promptly, and was gradually replacing BA.1 as dominant sublineage in several regions after a brief period. It also proved the quick evolution and mutation of Omicron variant at a high transmissibility, which efficiently spread to more than 190 countries/territories and here in China.^20^, ^21^ BA.3 had very limited community transmission capacity with at most a few hundred cases in the world.^22^ Since first found in South Africa in mid‐December 2021 (November 25, 2021 to January 01, 2022) and early January 2022 (December 10 2021 to February 06, 2022), respectively, BA.4 and BA.5 have circulated there as the dominant variants,^16^ and have promptly replaced BA.2, with more than half of the detected cases since April, 2022.^23^ A viral evolutionary biologist, Jesse Bloom, agreed that the spread of BA.4 and BA.5 was faster compared to other Omicron sublineages.^23^ ECDC indicated that BA.4 and BA.5 dominate the EU/EEA on September 2022 (Figure 4).^24^

**Figure 5:**
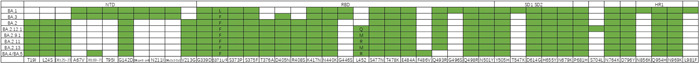
Spike mutations of Omicron five sublineages and four descendent lineages. Only include substitution and deletion changes

Recent emergence of Omicron descendent lineage BA.2.12.1 contains L452Q mutation and shows potential higher transmissibility compared to BA.2. BA.2.12.1, which has surged in some parts of North America, is able to evade antibodies elicited by having a previous Omicron infection, even after being vaccinated with a booster dose.^25^, ^26^ Currently, BA.2.12.1 dominates the United States, which is projected to be 62.2% of the national proportion, leading to the epidemic resurgence.^27^ And other Omicron descendent lineages, like BA.2.9.1, BA.2.11, and BA.2.13, share all S mutations of BA.2 and have an additional mutation (BA.2.9.1: L452M, BA.2.11: L452R, BA.2.13: L452M, respectively). BA.2.9.1 shares all S mutations with BA.2.13 and have 4 additional mutations outside the S (ORF1a: K332R, ORF3a: H78Y, N: P67S, N: S412I) (Figure 5).^28^, ^29^

## 3 OMICRON INFECTION IN CHINA

The first confirmed imported Omicron BA.1 case on the Chinese mainland was identified on December 9, 2021, with an international passenger from Poland to Tianjin. This asymptomatic carrier was under closed‐loop management/treatment and BA.1 was not spread domestically.^30^ The first confirmed imported BA.2 case on the Chinese mainland was identified on December 27, 2021, with an international passenger from Oman to Guangdong Province.^31^ In early‐January, 2022, the first‐ever locally‐transmitted Omicron BA.1 cases were confirmed and reached community transmission stage in Tianjin, Chinese mainland, and have a significant transmission superiority than prior Delta variant (https://view.inews.qq.com/a/20220109A03H MP00). However, the infection rate of BA.1 sublineage started to subside in the precise prevention and control. Following BA.1, the BA.2 sublineage could be the catalyst for the outbreak of new epidemic wave of SARS‐CoV‐2 to sweep the Chinese mainland with an unpresented speed from late February, 2022, and was escalated abruptly the domestic epidemic that subsequently displaced Delta variant and BA.1.^20^ Unfortunately, the Chinese mainland has reported more than 600,000 locally‐transmitted confirmed BA.2 cases until May 5, 2022. Most importantly, the current epidemic in China was marked by BA.2's extremely high infectivity, cryptical/clustered transmissibility in the communities, and has been able to disclose every case of infection. Additionally, the infectious trend of vaccinated individuals strengthened the risk of immune evasion of BA.2 sublineage and highlighted serious challenges in the public health and social measures. During this period, reverse transcription‐polymerase chain reaction has played a crucial role for accurate diagnosis of COVID‐19 in fever clinics and large‐magnitude screening within medium to high‐risk areas.^32^

Recently, the first confirmed imported BA.4 case on the Chinese mainland was identified on April 30, 2022, with an international passenger from Amsterdam to Guangzhou City, Guangdong Province, and the patient was fully immunized individual.^33^ The first confirmed imported BA.5 case on the Chinese mainland was identified on April 29, 2022, with an international passenger from Uganda to Shanghai Municipality, and the patient had been received four doses of Sinopharm COVID‐19 vaccine (Beijing Institute of Biological Products Co, Ltd).^34^ The first case confirmed imported BA.2.12.1 on the Chinese mainland was identified on April 27, 2022, with an international passenger from Kenya to Guangzhou City, Guangdong Province, and the patient had fully vaccination.^35^ Overall, vaccination did not maintain a high level of protection against Omicron variant due to “breakthrough” infections occurred in fully immunized individuals, who in turn spread SARS‐CoV‐2 to other individuals.^25^ These imported cases posed an added concern for public health and social measures. To date, a variety of public health policies worldwide have been implemented for managing the COVID‐19 pandemic according to the national conditions of various countries. Chinese government has implemented and optimized the “dynamic zero‐COVID policy” for effective combating Omicron.^36^, ^37^

## 4 TRANSMISSIBILITY AND SEVERITY OF THE OMICRON VARIANT

Epidemiologists analyze an epidemic's growth as well as transmissibility of Omicron variant using reproduction number, the average number of new infectious caused by each typical infection.^38^ Estimating the basic and effective reproduction number (R0 and Re) timely and accurately throughout a COVID‐19 pandemic is critical for tracking pandemic dynamics, and identifying the need and strategy to mitigate transmission, like social distancing.^39^ The epidemiological data manifested that the average R0 of WT and Delta were 3.28 (1.4–6.5) as well as 5.08 (3.2–8.0), respectively.^40^, ^41^

**Figure 6:**
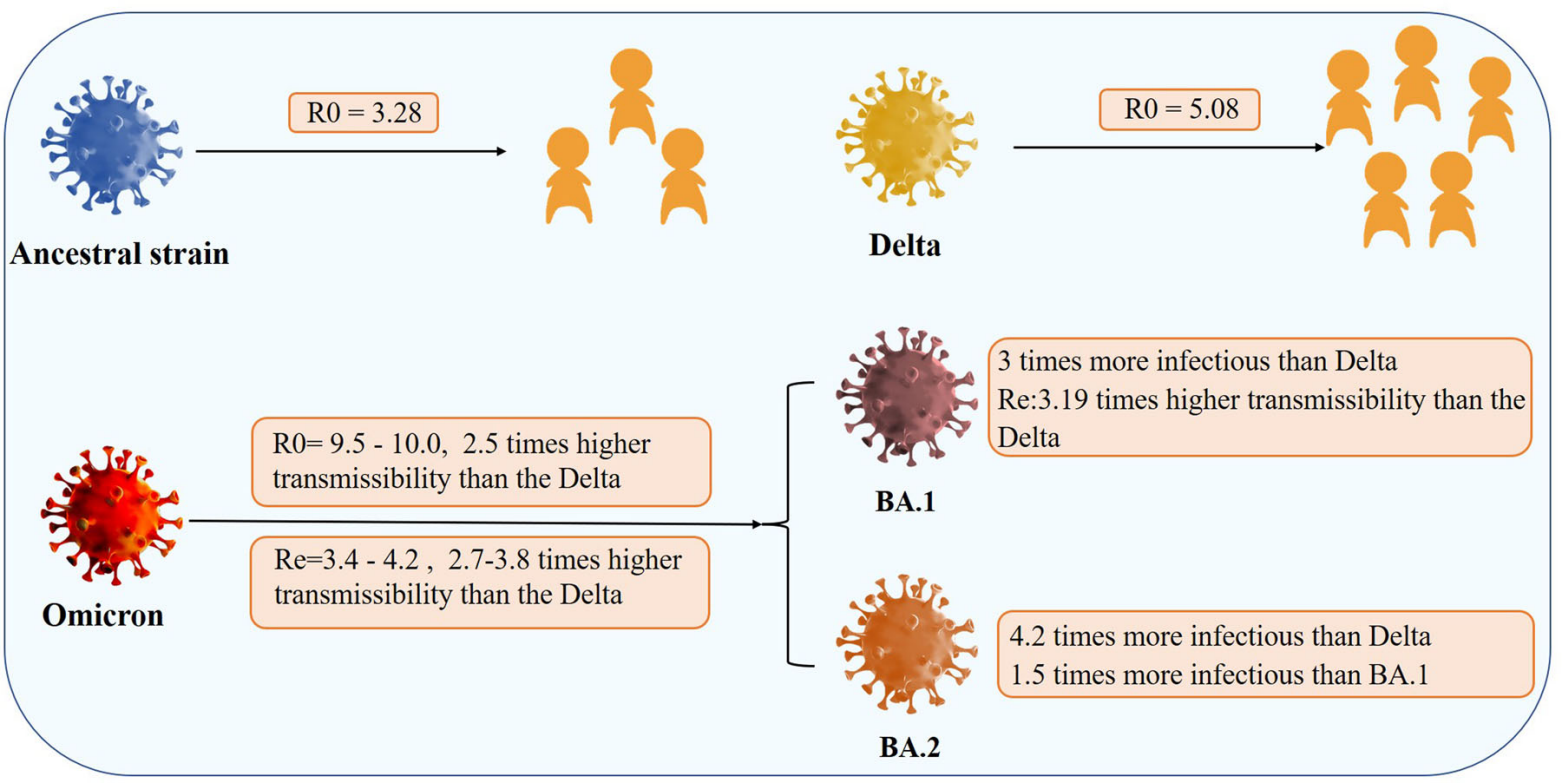
The basic and effective reproduction number (R0 and Re) of SARS‐CoV‐2

While, the average R0 for the Omicron variant was 9.5–10.0 (5.5–24.0),^42^, ^43^ that was, each infected person infected an average of 9.5–10 people. The average Re for Omicron was 3.4–4.2, 2.7–3.8 higher than for the Delta, while the its R0 exhibited 2.5 times higher transmissibility than Delta.^39^, ^42^ The infection curves were observed that Omicron variant grew significantly faster than Delta, with a doubling time between 1.2 and 3 days, making contact tracing difficult.^43^, ^44^ That was a huge advantage for Omicron, therefore medical workers had to rely on other measures, like daily nucleic acid testing. Since the advent of BA.2 sublineage, it had become the predominant epidemic strain worldwide with approximately 4.2 and 1.5 times as infectious as Delta and BA.1, respectively (Figure 6).^45^ These findings indicated that novel mutations in S protein influences infectivity of previously infected and vaccinated individuals, with the BA.2 sublineage showing more efficient ACE2‐mediated infection and immune evasion than WT or other variants. Several studies showed that the transmissibility of BA.4/5 and BA.2.12.1 was slightly faster than that of BA.2 by neutralization escape induced by both vaccination and infection.^24^, ^46^ Omicron BA 2.12.1 subvariant spread quickly, triggering a new surge in many parts of the United States and spreading to 66 countries and regions.^24^, ^47^, ^48^ Besides, research indicated that BA.5 had a growth advantage against other Omicron sublineages with a higher ability of immune escape than the BA.1‐3, and BA.2.12.1.^49^ So large numbers of people with antibody‐mediated natural immunity caused by earlier BA.1 and BA.2 or immune response derived by vaccination can still infect with BA.5.^50^ Recently, new surveillance data suggest that BA.5 sublineage is the catalyst for the new wave of the COVID‐19 epidemic outbreak to spike globally with an unpresented speed, and is rapidly dominating in several countries/regions.^51^, ^52^

Although the transmissibility was increased, a declined trend was observed in hospitalization and mortality rates during Omicron according to the data.^53^ For instance, in a retrospective cohort study, Nyberg et al.^54^ showed that compared with Delta, those with Omicron were at low risk of severe outcomes after SARS‐CoV‐2 infection with significant variation associated with age. Menni et al.^55^ reported a prospective longitudinal observational study involving 63002 SARS‐CoV‐2‐positive individuals and reported symptoms in the ZOE COVID Study app. The results showed that the main characteristics of Omicron infection was that the smell loss during Omicron wave was less common in participants with infection than during the Delta wave, sore throat was more often, and the hospitalization rate was lower. In fact, a recent research also supported that the viral replication in the lungs, pro‐inflammatory cytokine production and the progression of overall disease were significantly reduced with infection of Omicron.^56^ Other animal studies aligned with the findings showing less pathogenicity and severity for the upper and lower respiratory tract after inoculation with Omicron BA.1 and BA.2 compared to other SARS‐CoV‐2 VOCs in the mice and hamster models.^57^, ^58^, ^59^, ^60^ Furthermore, in hamster model, Tamura et al.^61^ showed that BA.5 exhibited still lower pathogenicity than the ancestral strain but gained high pathogenic by evolving to enhance severe inflammatory response when compared to BA.1 and BA.2. Consistent with this, Kimura et al.^62^ also indicated that the latest BA.4 and BA.5 subvariants might cause more severe lung disease than BA.2, and compared to L452R/M/Q‐bearing BA.2‐related Omicron variants, especially BA.4/5, had potentially greater risk of global health.

At the same time, COVID‐19 vaccines can further reduce the risk of infection, hospitalization and death in people. A meta‐analysis reported on the effectiveness of the booster dose, showing a 71% reduction in SARS‐CoV‐2 infection compared with those who did not receive a booster shot.^63^ An observational study assessing Hong Kong residents aged 20 years and older suggested that severe disease and death occurred predominantly in the unvaccinated population and two doses of BNT162b2 provided a very high level (>85%) of protection against severe disease and death in all ages.^64^ Meanwhile, another study in Israel also suggested that a third dose was estimated to be 93%, 92%, and 81% effective in preventing COVID‐19‐related hospitalizations, preventing severe illness and preventing COVID‐19‐related death, respectively, as compared with two doses administered at least 5 months ago.^65^ By contrast, adding a fourth dose of an mRNA COVID‐19 vaccine reduced premature mortality from all causes among in the oldest and frailest individuals.^66^ A study from Canada showed an increase in effectiveness against any SARS‐CoV‐2 infection (19%), symptomatic infection (31%), and severe outcomes (40%) related to the omicron variant among older residents with long term care who received the fourth dose of mRNA vaccine compared with those who received a third dose 84 or more days previously.^63^

## 5 VACCINES AND TREATMENT

The highly contagious Omicron variant along with the excess number of nonsynonymous S mutations, which was related to increase transmissibility and superior immune evasion, raised concerns about the effectiveness of current vaccines and antibody therapeutics.^67^ Liu et al.^68^ identified S371L, N440K, G446S, and Q493R mutations that confer greater antibody resistance on Omicron, and thus threaten the efficacies of current vaccines. Studies have shown that the efficacy of the vaccine is associated with neutralization antibody levels. The higher the level of neutralization antibodies, the more effective the vaccine is, which supports that neutralization antibody levels can be used to predict vaccine efficacy.^69^, ^70^ Preliminary experimental data from multiple studies were used to evaluate the ability of current vaccine protection. Lusvarghi et al.^71^ estimated neutralization of BA.1 pseudoviruses by postvaccination serum samples after two and three doses vaccination, the results found that neutralizing antibody titer were lower following two doses of vaccine or in many convalescent serum samples. Most vaccines were developed before the emergence of Omicron, which exhibited marked resistance and escape from neutralization by monoclonal antibodies and sera from vaccinated individuals.^71^, ^72^

**Figure 7:**
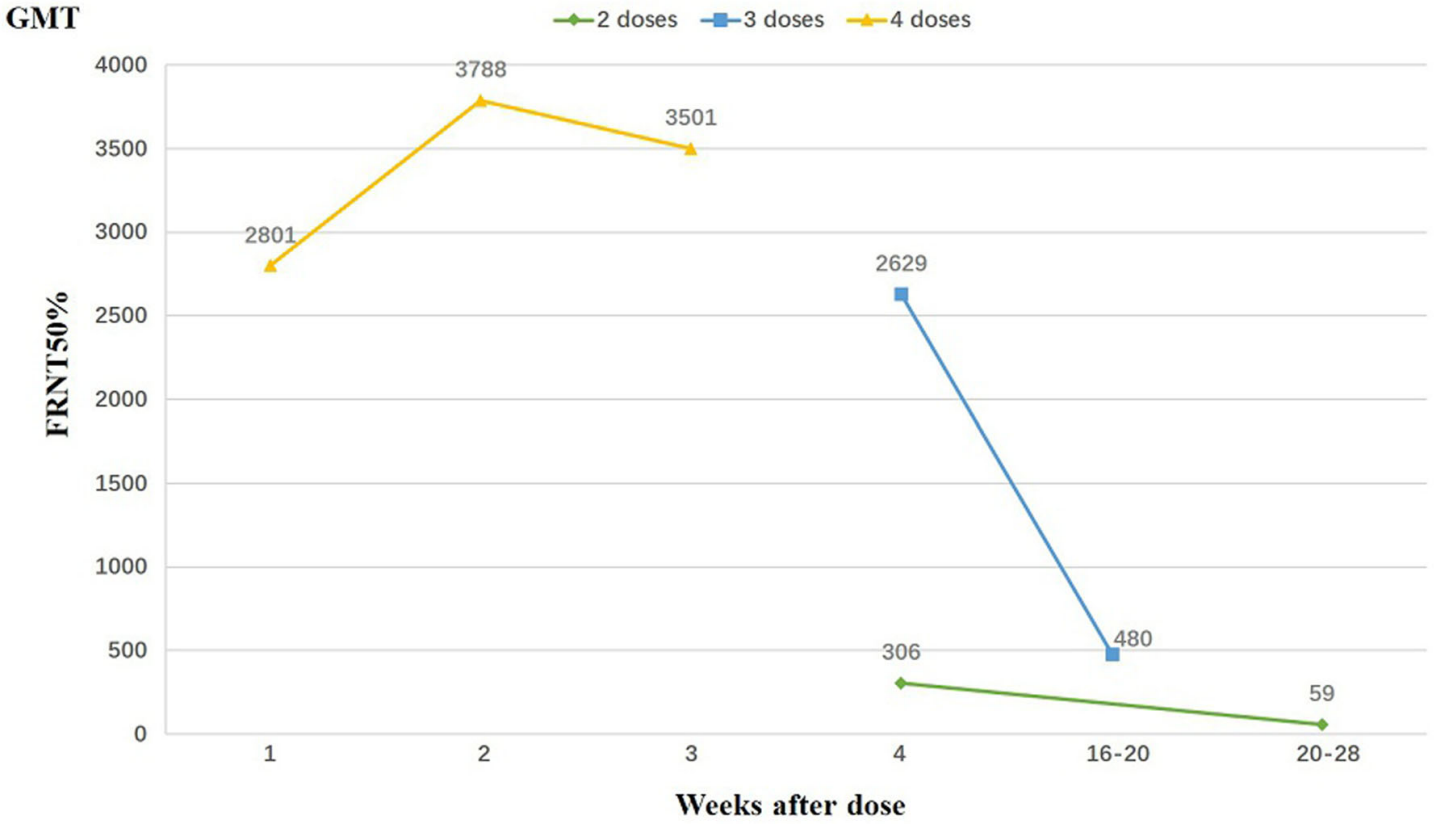
Neutralizing antibody titers after receipt of two, three and four doses of BNT162b2. FRNT, focus reduction neutralization test; GMTs, geometric mean titers.

Ai et al.^73^ reported that serum from two doses of an inactivated whole‐virion vaccine (BBIBP‐CorV) recipients showed weak neutralization activity against BA.1, BA.1.1, BA.2 and BA.3 sublineages, while the neutralizing activities against Omicron sublineages were maintained at a relatively higher level following the third booster dose of homologous inactivated vaccine (BBIBP‐CorV) or heterologous protein subunit vaccine (ZF2001), which suggested the importance of COVID‐19 vaccine booster dose. In addition, an efficacy clinical intervention trial analysis of the fourth dose mRNA vaccines, BNT162b2 or mRNA1273, showed that the booster dose restored titers of neutralizing antibodies (Figure 7). The urgency for next generation vaccine development has been highlighted by the low efficacy in preventing mild or asymptomatic Omicron infections or the breakthrough cases.^74^ Latest evidence by WHO on 1 June, suggested that “hybrid immunity” (combinations of vaccine) against severe COVID‐19 was more robust among than infection‐induced or vaccine‐induced immunity alone. The vaccination to cover all the population should be high priority from public health authorities, WHO said.^75^ Therefore, popularization of booster vaccination regularly per year is still a safe and effective strategy to prevent SARS‐CoV‐2 infections. Most importantly, heterologous booster is recommended for sequential vaccination, which can induce the body's immune system to produce effective “cross‐immune memory” or “complementary‐immune memory” so as to maximize protection against COVID‐19 severe disease and death.^76^

To be clear, Omicron is a potential reference strain for the development of next generation COVID‐19 vaccines, due to a large number of S mutations. Fortunately, besides the available vaccines and heterologous vaccination approach, a Phase Ⅱ/Ⅲ clinical trials (NCT04927065) reported well immunogenicity data and safety for first bivalent COVID‐19 vaccine booster candidate, mRNA1273.211. Compared to mRNA‐1273, there was a 2.20‐fold and 2.15‐fold increase respectively in the neutralizing antibody titers against Omicron with the mRNA‐1273.211 booster dose (8.7‐9.7 months interval after the first shot) at 1 month and 6 months after administration. Data indicated that bivalent booster vaccines induced potent and durable immune protection providing a critical mitigation tool for emerging sublineages.^77^ In the meantime, the domestic new vaccines specifically targeting Omicron variant that have been approved by NMPA to enter the clinical trial stage include the second‐generation recombinant protein subunit COVID‐19 vaccine (NVSI‐06‐08) (Chinese Academy of Biological Sciences) on April 3, whole inactivated virus particle vaccine (Beijing Institute of Biological Products Sinopharm) on April 26, and COVID‐19 mRNA vaccine (Stemirna Therapeutics, Ltd) on April 29, which will soon be available for emergency use. In addition, for longer‐term considerations, some non‐profits, government agencies and vaccine makers have made the development of pan‐SARS‐CoV‐2 or pan‐sarbecovirus vaccines as a research priority.^78^

On April 21, 2022, WHO provided two new recommendations for COVID‐19 mild patients, including a strong recommendation and a conditional recommendation for use of therapeutic agent Paxlovid and remdesivir respectively in patients during highest hospitalization risk.^79^ Meanwhile, Paxlovid was been used to hospitalized COVID‐19 patients in China, with the hope of reducing hospitalization time and death rates.^20^ An oral nucleoside drug VV116 displayed boost anti‐SARS‐CoV‐2 benefit in preclinical studies. Shen et al.^80^ evaluated safety and effectiveness of VV116 in Chinese Omicron subjects from March 8, 2022 to March 24, 2022. The data showed that VV116 exhibited safety and effectiveness in subjects, and SARS‐CoV‐2 nucleic acid shedding time was shortened by 2–3 days. Evusheld, a combination of the two long acting antibodies tixagevimab and cilgavimab, is authorized by FDA for prevention and protection against infection with a dose of 300 mg of tixagevimab and 300 mg cilgavimab on June 29, 2022.^81^, ^82^ FDA continues to monitor the neutralizing activity of Evusheld against emerging SARS‐CoV‐2 variants and will provide additional updates as needed. Takashita et al.^83^ reported that the three small‐molecule antiviral drugs remdesivir, molnupiravir (inhibitors of the RNA‐dependent RNA polymerase of SARS‐CoV‐2), and nirmatrelvir (an inhibitor of the main protease) might have therapeutic value against BA.2.12.1, BA.4, and BA.5, and also indicated that bebtelovimab was effective against BA.2.12.1, BA.4, and BA.5.

## 6 CONCLUSIONS

Clearly, COVID‐19 pandemic will continue. There are a number of subvariants that WHO is tracking, for example, the emerging new BA.5 sublineage BA.5.2.1.7 (BF.7) is circulating at an incredibly intense level around the world, and could be the next dominant variant.^84^ By and large, the next SARS‐CoV‐2 VOC including possible SARS‐CoV‐2 recombinant will appear. Therefore, there is still a long way to go, as we live in an interconnected world. Currently, protecting strategies have been dramatically changed worldwide, the restrictions imposed due to the COVID‐19 epidemic have been lifted in many countries, however, the number of severe case and death has not shown a sharp increase wave. To enhance the medical countermeasures to rapidly respond to the emerging Omicron sublineages and future VOCs, the best way demands the continuous update and development of effective vaccines, antivirals, rapid molecular diagnostic tools, and in combination with keeping surveillance activities up, to play a decisive role. The key is that there is a need to rapidly achieve up global vaccination coverage rates, thereby preventing possible future outbreaks.

## AUTHOR CONTRIBUTIONS

Yan Wu performed the original draft preparation, wrote review. Feng Wang wrote review. Yiyin Long wrote review and editing. Wei Liu contributed supervision, writing – review and editing. Yuliang Wang contributed supervision, writing – review and editing.

## CONFLICT OF INTEREST

The authors declare no conflict of interest.
